# 

**Published:** 1915-09-18

**Authors:** 


### CONTENTS.

PAGE

**The Medical Needs of the Army and of the Hospitals** ... ... ... ... ... ... 513

**Of Functional Nervous Disease** ... ... ... 514

**Hospital and Institutional News** ... ... ... 515

An Old Hospital Rebuilt—Death Dangers of Disunion—Praise for an Asylum Staff—The Retention of a Doctor's Fee—Killing Two Birds with One Stone—The L.G.B., the Guardians, and Economy—A Mental Hospital Superintendent and the War—Operations on Non-Paupers at Poor-Law Infirmaries—An Electrophone Bullet Probe—Why are Workmen's Subscriptions Withheld? — An Unreasonable Board of Guardians—Local Government in Scotland—Twenty-Five Years' Progress—The Notification of Measles—Interfering between Practitioner and Patient—A Panel Doctor and His Patients —Great Gift for Cardiff Hospital—Sanitary Problems in Hospitals for Indians—How Caste Prejudices were Met—Another Drain on the Funds—Hospitals and the Spirit Tax—Hospitals on Wheels—Medico-Legal Dangers of Silver Nitrate—Eye Defects and the Fighting Line—An Episode in the History of the Society of Apothecaries—This Week's Drug Market.

**In a General Hospital** ... ... ... ... 521

Possibly Abortive Cerebro-Spinal Fever.
Case Notes of an Obscure Disease.

PAGE

**The Welsh Anti-Tuberculosis Campaign** ... 523

The Measure of the Memorial Association's Activities.

**Post-Graduate Education and the War** ... 525

The Need of More Facilities for Post-Graduate Study.

**National Insurance Act Developments** ... ... 526

Some Points from Recent Circulars.

**The Metropolitan Asylums Board Report** ... 527

1914 a Strenuous Year.

**The Late Mr. Frederick Lawrance** ... ... 528

**A Profitable Hospital Garden** ... ... ... 529

**Buildings Contemplated and in Progress** ... 530

**Round the World** ... ... ... ... ... ... 531

Fellow-Workers and the Roll of Honour.

**Medical Appointments** ... ... ... ... 532

**Personalia** ... ... ... ... ... ... 532

**Norfolk War Hospital** ... ... ... ... 532

**Gifts and Bequests** ... ... ... ... ... 532

**Latest Installations in Hospital Equipment** ... 532

**The Institutional Worker** ... ... (Suppl.) 1

The Purchase and Maintenance of Surgical Instruments and Appliances.
Enquiries and Answers.
The Sick and In Need.
Employment and Training.

